# Supplementary material for: Polysaccharide-Stabilized Semisolid Emulsion with Vegetable Oils for Skin Wound Healing: Impact of Composition on Physicochemical and Biological Properties
Source: Pharmaceutics. 2024 Nov 8;16(11):1426. doi: 10.3390/pharmaceutics16111426 (PMC11597777; doi:10.3390/pharmaceutics16111426)
Supplement: Supplementary file 1 [file pharmaceutics-16-01426-s001.zip › pharmaceutics-3277293-supplementary.pdf]

pharmaceutics

MDPI

Research Article – Supplementary file

Polysaccharide-Stabilized Semisolid Emulsion with Vegetable Oils for Skin Wound Healing: Impact of Composition on Physicochemical and Biological Properties.

Giovanna Araujo de Morais Trindade [1•], Laiene Antunes Alves [1•], Raul Edison Luna Lazo [1], Kamila Gabrieli Dallabrida [2], Jéssica Brandão Reolon [2], Juliana Sartori Bonini [2], Karine Campos Nunes [3], Francielle Pelegrin Garcia [3], Celso Vataru Nakamura [3], Fabiane Gomes de Moraes Rego [4], Roberto Pontarolo [1], Marcel Henrique Marcondes Sari [4*], Luana Mota Ferreira [1*].

[1] Centro de Estudos em Biofarmácia, Departamento de Farmácia, Programa de Pós-Graduação em Ciências Farmacêuticas, Universidade Federal do Paraná, Curitiba-PR, 80210-170, Brasil; giovannaaraujo@ufpr.br; laienealves@ufpr.br; raulunalazo@gmail.com; pontarolo@ufpr.br; luanamota@ufpr.br.
[2] Departamento de Farmácia, Universidade Estadual do Centro-Oeste, Guarapuava-PR, 85040-167, Brasil; kadallabrida@gmail.com; jessica_breolon@yahoo.com.br; juliana.bonini@gmail.com.
[3] Laboratório De Inovação Tecnológica No Desenvolvimento De Fármacos E Cosméticos, Universidade Estadual de Maringá, Maringá – PR, 87020-900, Brasil; fpgarcia2@uem.br; kaahnunes07@gmail.com; cvnakamura@uem.br.
[4] Departamento de Análises Clínicas, Universidade Federal do Paraná, Curitiba-PR, 80210-170, Brasil; rego@ufpr.br; marcelsari@ufpr.br.
* Correspondence: MHMS, marcelsari@ufpr.br; LMF, luanamota@ufpr.br.
• These authors contributed equally to this work

Citation: To be added by editorial staff during production.

Academic Editor: Firstname Lastname

Received: date
Revised: date
Accepted: date
Published: date

Copyright: © 2024 by the authors. Submitted for possible open access publication under the terms and conditions of the Creative Commons Attribution (CC BY) license (https://creativecommons.org/licenses/by/4.0/).

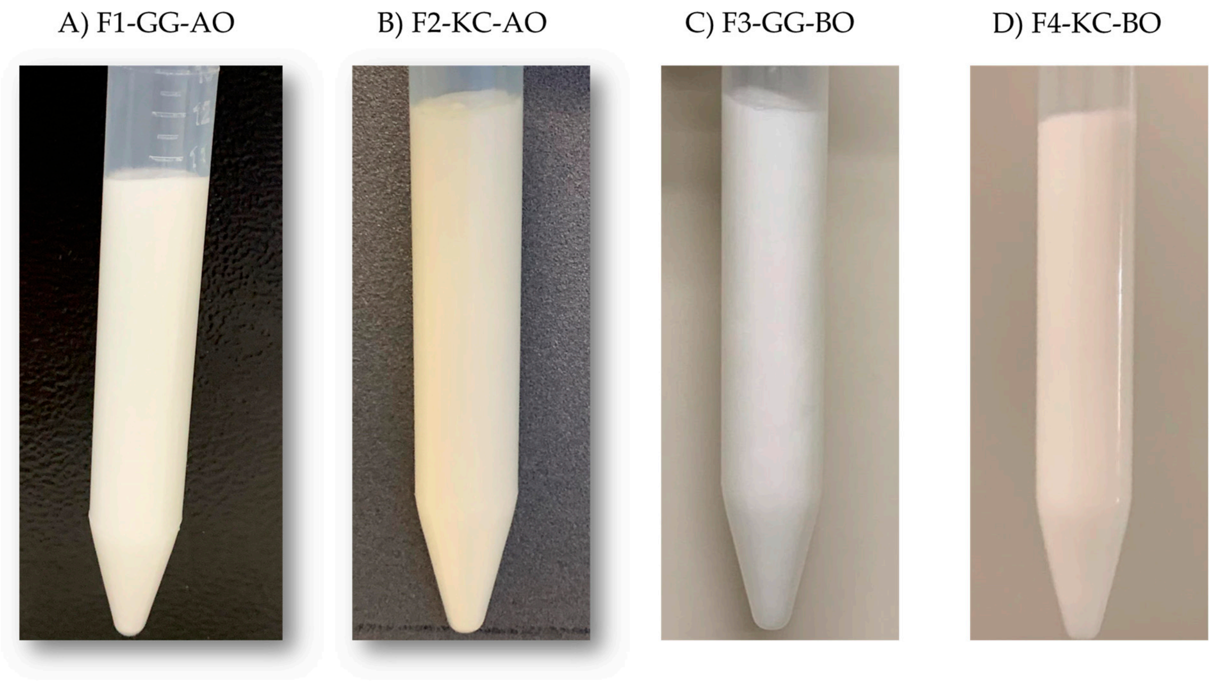

Figure S1. Images of semisolid formulations after centrifugation test.

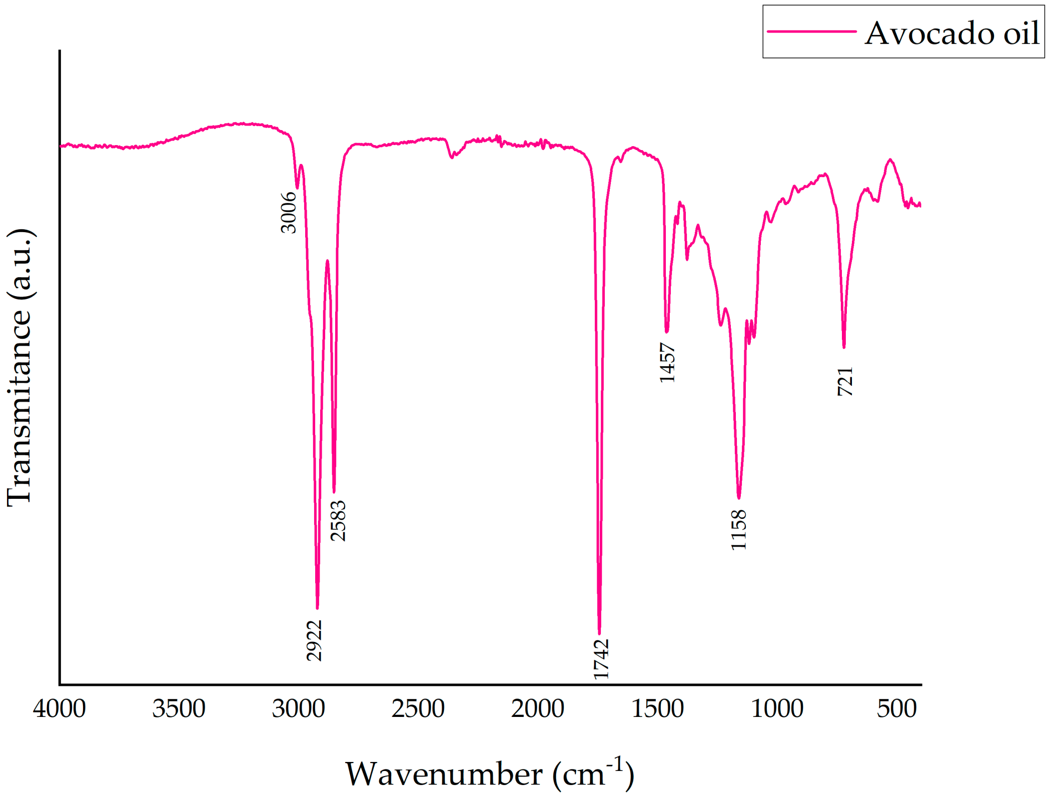

Figure S2. Infrared spectra of avocado oil.

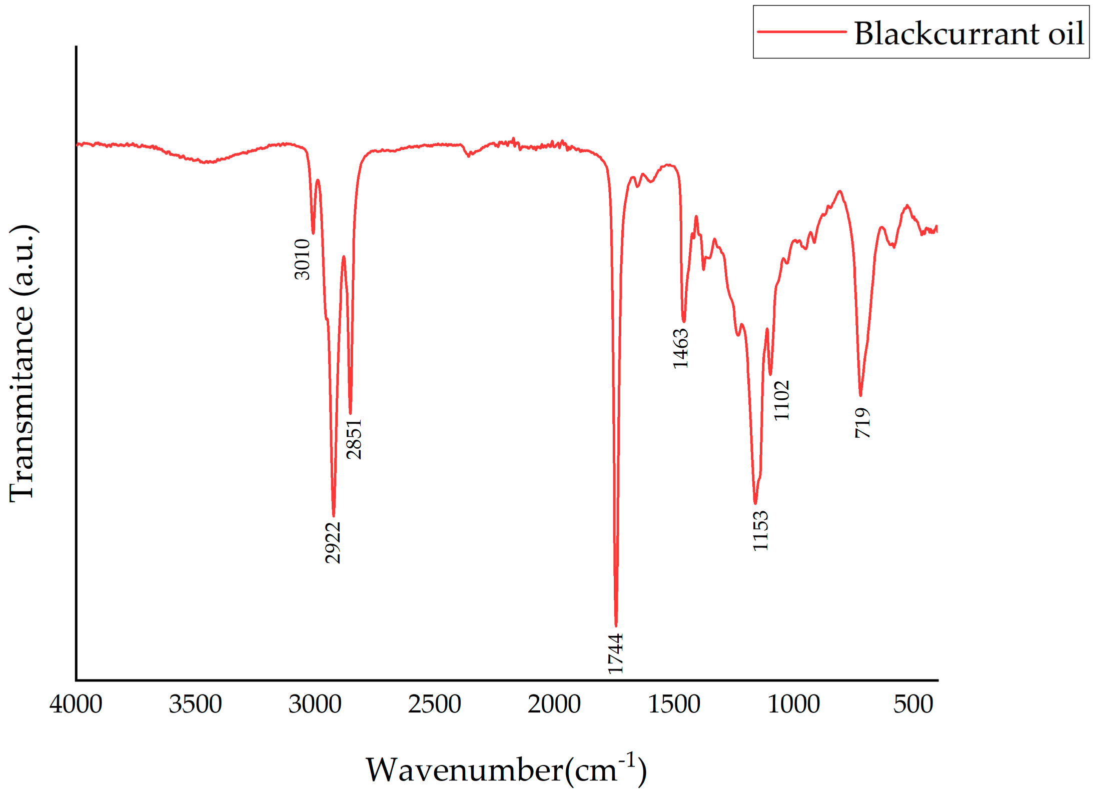

Figure S3. Infrared spectra of blackcurrant oil.

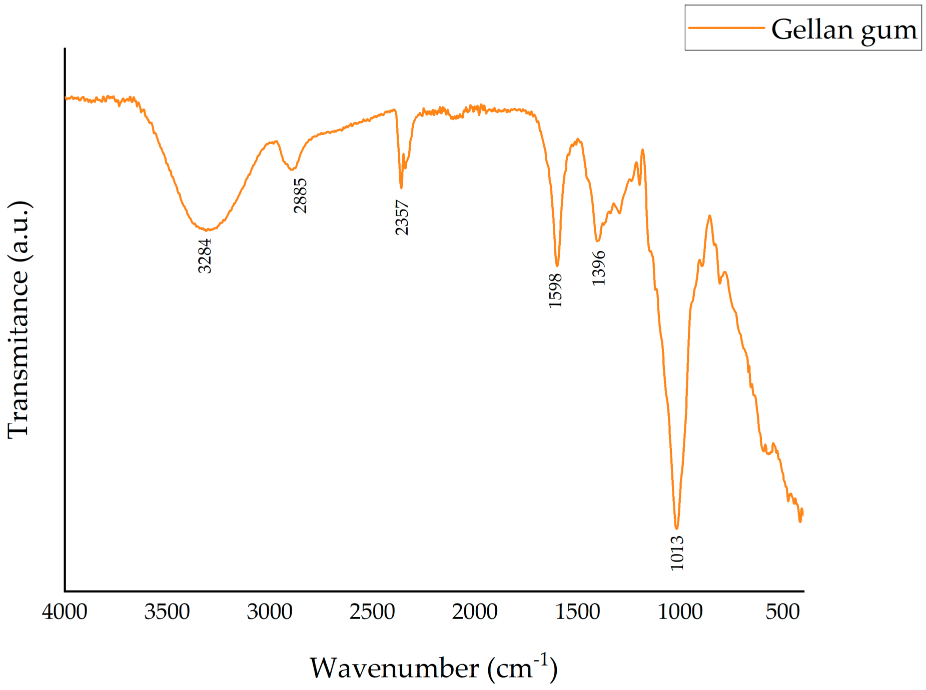

Figure S4. Infrared spectra of gellan gum.

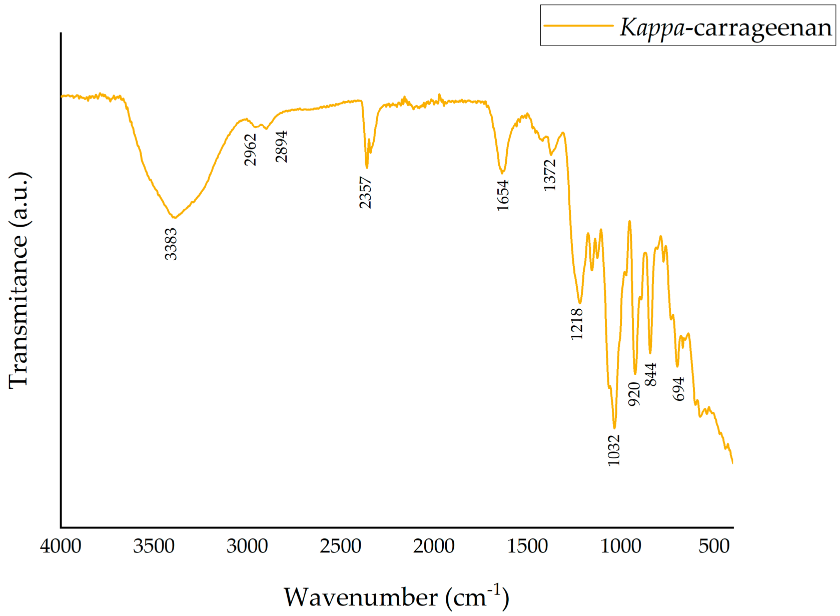

Figure S5. Infrared spectra of *kappa*-carrageenan.

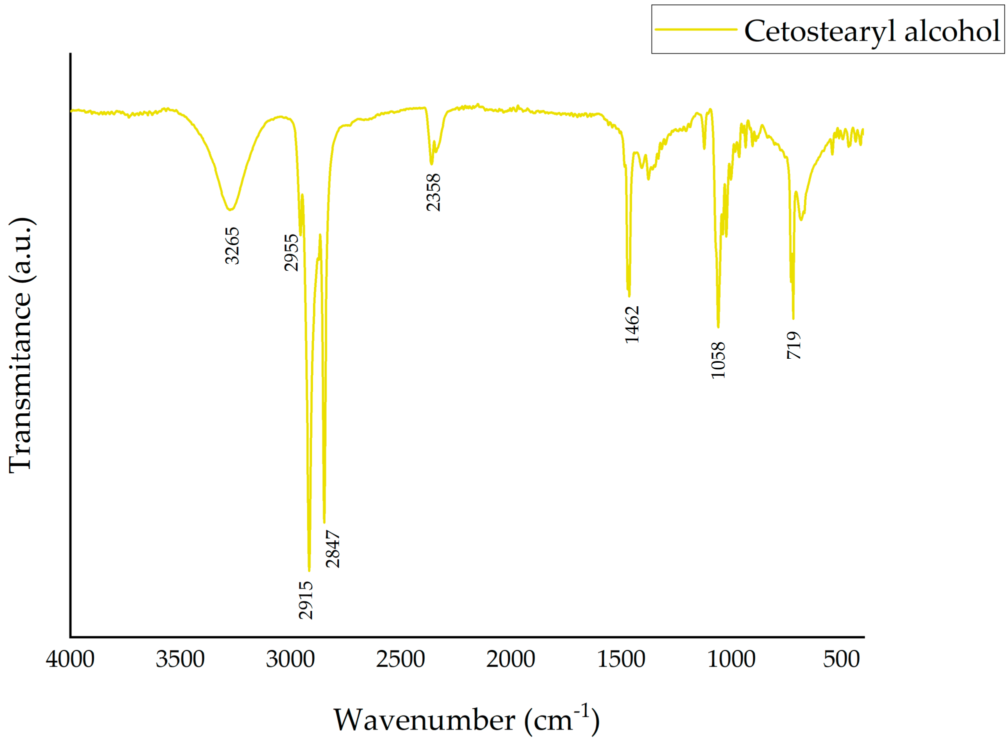

Figure S6. Infrared spectra of cetostearyl alcohol.

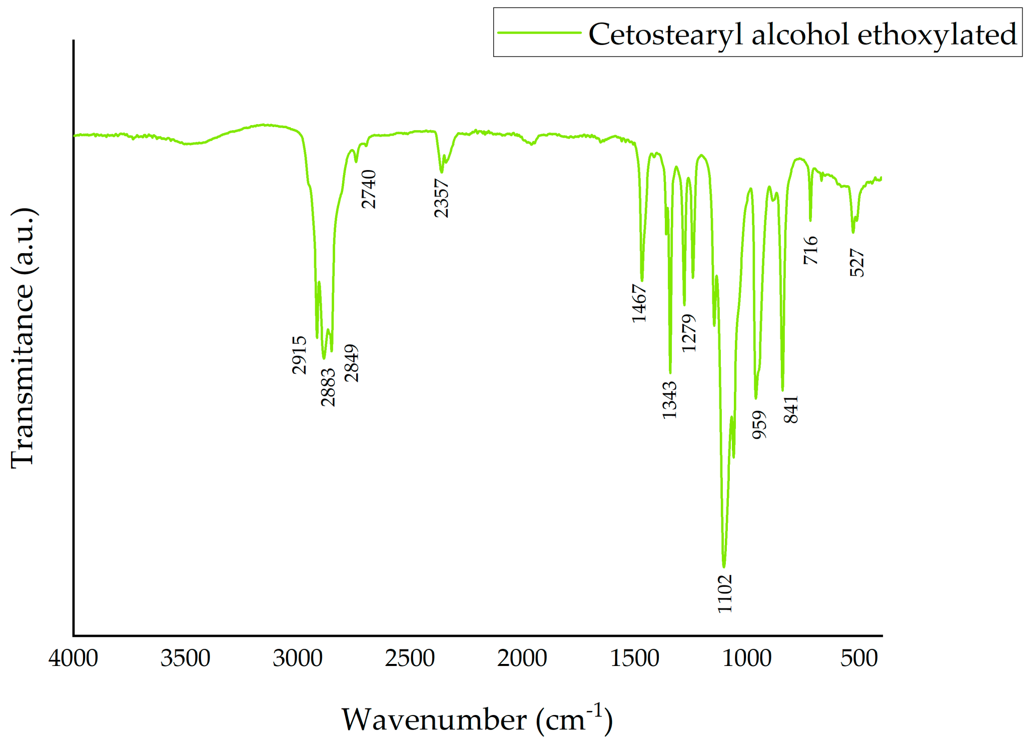

Figure S7. Infrared spectra of cetostearyl alcohol ethoxylated.

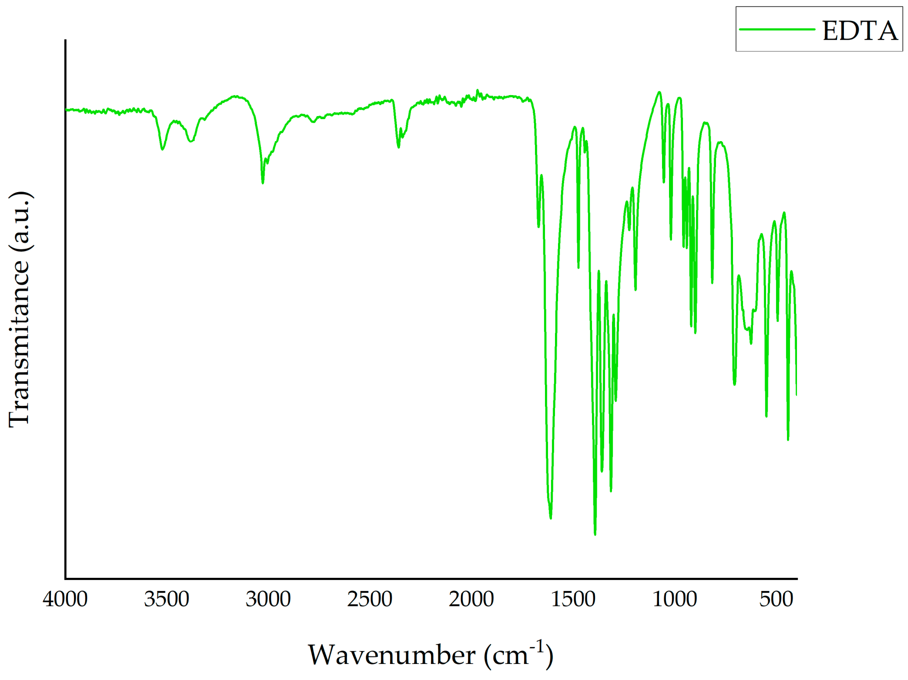

Figure S8. Infrared spectra of EDTA.

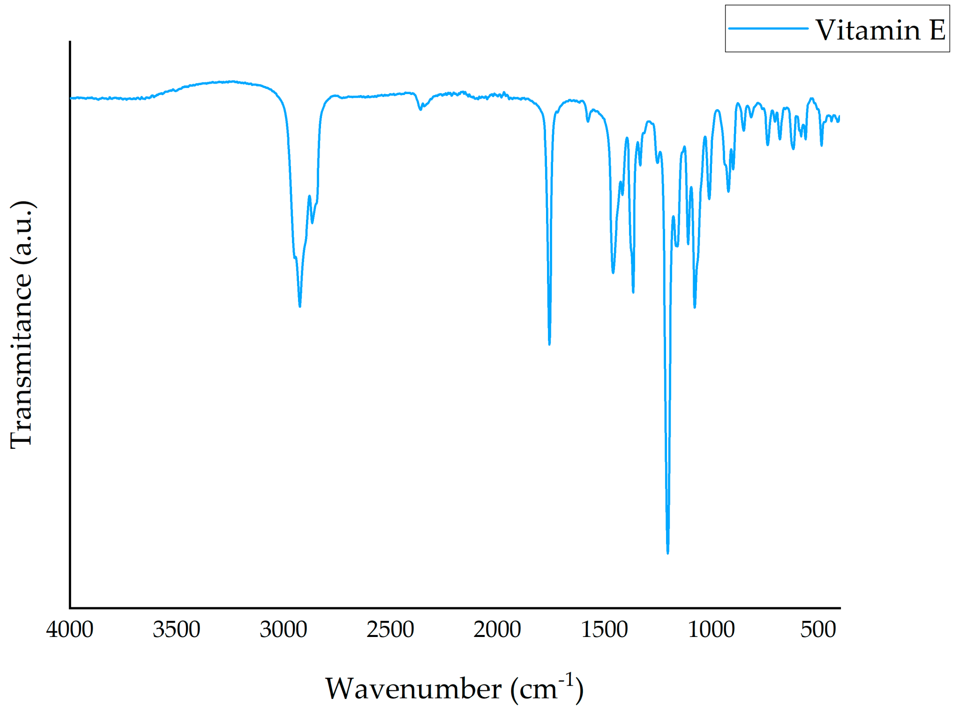

Figure S9. Infrared spectra of Vitamin E.

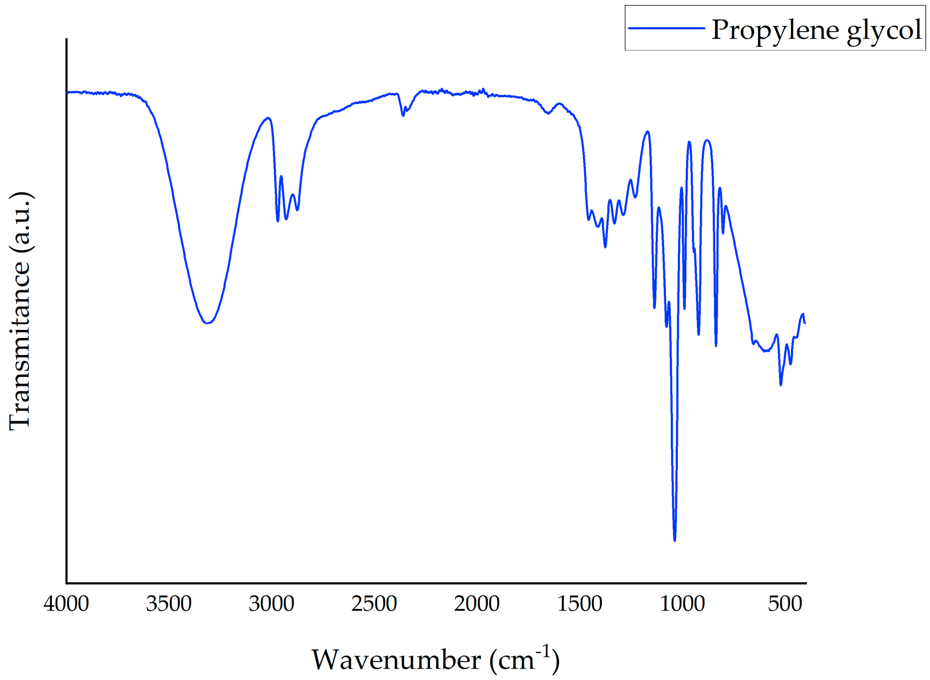

Figure S10. Infrared spectra of Propylene glycol.

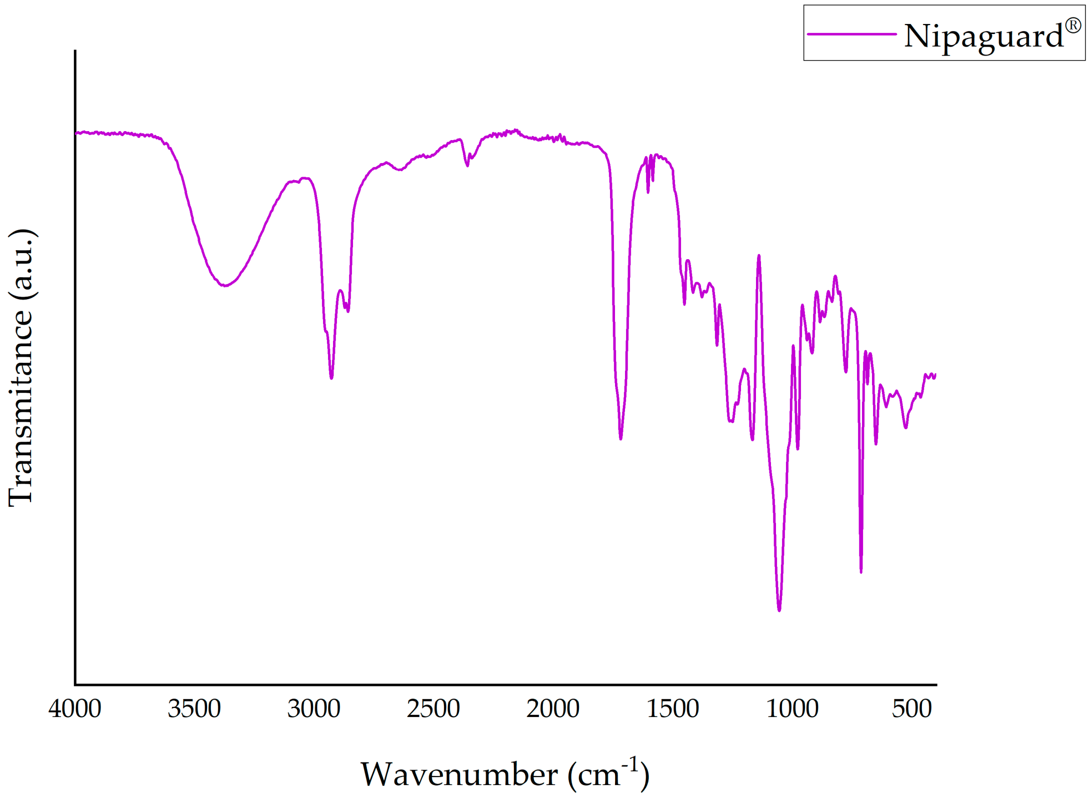

Figure S11. Infrared spectra of Nipaguard®.
